# Efficacy of a brief online intervention in reducing excessive worry and improving daily functioning: A randomized trial with mediation analysis

**DOI:** 10.1016/j.invent.2025.100842

**Published:** 2025-06-10

**Authors:** Tove Wahlund, Fredrik Spångberg, Viktor Vadenmark, Erik Andersson

**Affiliations:** Division of Psychology, Department of Clinical Neuroscience, Karolinska Institutet, Stockholm, Sweden

## Abstract

Excessive worry is common among treatment-seeking individuals in primary care and has a negative impact on daily functioning, which may also lead to other mental health problems. The current study tested whether a worry-focused online intervention – provided in both a guided and an unguided format – was efficacious in reducing worry-related symptoms and if these effects were specifically linked to improvements in daily functioning. A total of 82 participants were randomized to intervention with therapist support (guided; *n* = 28), intervention without therapist support (unguided; *n* = 27) or to waiting list (n = 27). Results showed that the online intervention was more effective than waiting list in reducing worry at week 5 (between-group *d* = 0.96). The intervention was effective against waiting list irrespective of whether it was provided in a guided (between-group *d* = 0.90) or unguided format (between-group *d* = 1.07) with sustained results at the 7-week follow-up. Reduction in worry mediated improvement in daily functioning (between-group *d* = 0.58; indirect effect estimate = −1.06 [95 % CI: −1.76 to −0.51], 66 % mediated effect). The mediation effects were fairly robust to mediator-outcome confounding, with residual correlation values set to *r* = 0.3 in a sensitivity analysis. The results provide further evidence that it is beneficial to provide a low-threshold, easy access intervention to patients with excessive worry, irrespective of primary diagnosis. Clinical implications are discussed.

## Highlights

• This trial showed that a brief online intervention was effective in reducing degree of excessive worry.
• The intervention reduced worry both in guided- and unguided format.
• Reductions in excessive worry mediated improvements in daily functioning.

## 1 Introduction

Excessive worry is defined as recurrent, intrusive thoughts about potential future negative events (Gonçalves and Byrne, 2013). Elevated levels of worry is the core symptom in generalized anxiety disorder (GAD; American Psychiatric Association, 2013) which is a common psychiatric disorder in treatment-seeking individuals in primary care (Wittchen and Hoyer, 2001). Previous research has shown that there is an additional large population with subthreshold levels of GAD who have levels of distress and psychosocial impairment comparable to those of individuals with a GAD diagnosis (Haller et al., 2014). Excessive worry may not only lead to GAD but has been found to contribute to other mental health problems such as symptoms of post-traumatic stress disorder (PTSD), depression, prolonged grief, insomnia, eating disorders, stress, somatic health complaints and persecutory delusions (Butler et al., 1995; Davies et al., 2016; Eisma et al., 2017; Freeman et al., 2015; Hong, 2007; Jansson and Linton, 2006; Roussis and Wells, 2008; Sala and Levinson, 2016; Verkuil et al., 2012). Excessive worry has also been linked to reduced cognitive performance and impaired everyday functioning (Crowe et al., 2007; de Vito et al., 2019; Eysenck and Calvo, 1992; Hayes et al., 2008; Leigh and Hirsch, 2011; Pietrzak et al., 2012; Rapee, 1993; Stout et al., 2015; Trezise and Reeve, 2016; Zainal and Newman, 2021). This highlights the need for scalable and easily accessible interventions for this large group of individuals who struggle with bouts of excessive worry across different symptom presentations.

Some research studies have investigated fully automated interventions (i.e., interventions without any therapist support) for GAD and excessive worry. Dear et al. (2015) conducted a randomized trial for individuals with GAD (*N* = 338) and found that an unguided version of online cognitive behavior therapy (CBT) led to similar symptom reductions as therapist-guided CBT. In a trial for individuals with GAD (*N* = 230), Hirsch et al. (2021) showed that an unguided online bias modification program had positive effects on interpretation bias for individuals with GAD, which in turn mediated reductions in excessive worry with overall moderate effect sizes. A study by Joubert et al. (2021) showed large reductions in excessive worry and associated symptoms in 26 adults with elevated levels of repetitive negative thoughts who received a completely unguided online intervention. However, a subsequent randomized trial (*N* = 137) did not fully support these findings (Joubert et al., 2023).

In 2020, our research group developed a 3-week unguided online intervention for individuals who struggled with excessive worries related to the COVID-19 pandemic. Results from a randomized trial (*N* = 670) showed that the intervention produced significantly larger reductions in symptoms of excessive worry than a waiting list control group (between-group *d* = 0.74). The intervention also resulted in significant improvements in secondary outcomes (mood, daily functioning, insomnia, and intolerance of uncertainty), compared to the waiting list (Wahlund et al., 2021). A mediation analysis showed that reductions in excessive worry mediated improvements in daily functioning, i.e., people improved their ability to attend to and concentrate on work, school, or leisure activities, specifically due to the reductions in worry from the intervention (Andersson et al., 2024). That study, however, was conducted during the first months of the COVID-19 pandemic and it is unclear if the results extend to other forms of worry.

The aim of the current study was to test whether our brief, unguided worry-focused intervention was also effective for general worries in the current post-pandemic era. First, we wanted to compare the online worry-focused intervention against a waiting list control group. Second, we wanted to investigate if the intervention was effective in both guided and unguided formats. Third, we wanted to replicate previous findings and test whether reductions in worry mediated improvements in daily functioning, as suggested by previous research.

## 2 Methods

This was a randomized controlled trial where participants were allocated to the online intervention (supported by a therapist or delivered in an unguided format) or to a waiting list. The national ethical review board in Sweden approved the study protocol (registration ID: 2021–04747; the protocol was pre-registered at https://osf.io/hgdxr/). The trial was powered to detect a medium effect-size (*d* = 0.6–0.7; guided intervention vs. waiting list and unguided intervention vs. waiting list) and the target sample was therefore set to 90 participants (90 % power, one-tailed test). The study was not specifically powered to find mediation effects but a post hoc analysis, using the data from the trial, showed a power estimate of 52 %.

### 2.1 Study procedures

Participants were recruited via advertisements on social media and on the university's news bulletin board and were included in the study from February 4th to March 6th, 2022. Applicants registered on a study website where they received written information about the study and submitted their application for participation, including written informed consent. They also completed a screening of symptoms with the Generalized Anxiety Disorder Scale-7 (GAD-7), Montgomery Åsberg Depression Rating Scale – Self report (MADRS-S), Penn State Worry Questionnaire (PSWQ), Alcohol Use Disorders Identification Test (AUDIT), and Drug User Disorders Identification Test (DUDIT) and provided demographic information (sex, age, civil status, occupational status, educational attainment, any psychiatric and somatic disorders) and a description of their worry and its impact on their daily lives. Eligible individuals who did not fulfill any exclusion criteria were contacted by telephone for an assessment of eligibility and a diagnostic interview. Telephone screenings were conducted by two master level students in psychology (the second and third authors), under supervision by the first and last authors of the study. During the interview, inclusion and exclusion criteria were assessed, and the MINI International Neuropsychiatric Interview (Sheehan et al., 1998) was administered to assess the occurrence of any psychiatric disorders. The decision about study inclusion was made by the principal investigator of the study (the last author) when each participant had completed the baseline assessment. Participants were randomized via www.random.org by an individual blinded to the study design and hypotheses. The controlled intervention period lasted for five weeks. Participants randomized to waiting list were subsequently provided the intervention after having completed the post-intervention assessment.

### 2.2 Participants

**Fig. 1:**
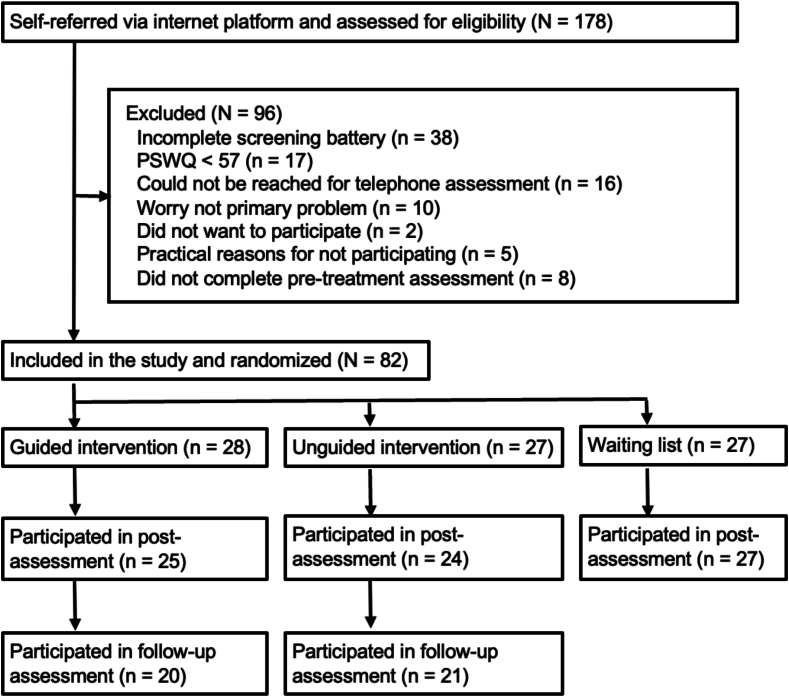
Study flowchart.

Fig. 1 shows the study flow. The study included 82 participants aged 18–72 years. Table 1 presents demographic and clinical characteristics of the sample. Inclusion criteria in the study were a) self-reported excessive worry, defined as a score ≥ 57 on the Penn State Worry Questionnaire (PSWQ; Meyer et al., 1990), b) at least one negative consequence of excessive worry (i.e., trouble sleeping, constantly checking news and/or social media, difficulties concentrating on other things, marked loss of productivity, or difficulties finding joy in everyday moments), c) ≥18 years of age, d) residing in Sweden, e) regular access to a computer or other device with internet connection. Exclusion criteria were a) non-Swedish speaking, b) severe depression or high suicide risk, and c) other principal diagnosis that could hinder participation in treatment (e.g., ongoing psychosis, alcohol- or substance use disorders).

**Table 1:** Demographic and clinical characteristics of participants.

Variable		Guided intervention (*n* = 28)	Unguided intervention (*n* = 27)	Waiting list (n = 27)
		M (SD)	M (SD)	M (SD)
Age	Mean Age (*SD*)	38.9 (11.0)	35.6 (8.3)	39.6 (14.8)
Sex	Female	25 (89.3)	23 (85.2)	24 (88.9)
	Male	2 (7.1)	4 (14.8)	2 (7.4)
	Other	1 (3.6)	0 (0)	1 (3.7)
Civil status	Married or registered partnership	11 (39.3)	7 (25.9)	5 (18.5)
	Cohabitation	9 (32.1)	15 (55.6)	10 (37)
	Living alone	8 (28.6)	3 (11.1)	11 (40.7)
	Other	0 (0)	2 (7.4)	2 (7.4)
Occupational status	Working full time	15 (53.6)	11 (40.7)	15 (55.6)
	Working part time	7 (25)	9 (33.3)	5 (18.5)
	Sick leave	2 (7.1)	2 (7.4)	2 (7.4)
	Student	4 (14.3)	6 (22.2)	4 (14.8)
	Retired	0 (0)	0 (0)	1 (3.7)
	Unemployed	2 (7.1)	2 (7.4)	2 (7.4)
	Disability pension	1 (3.6)	0 (0)	0 (0)
Highest level of education	Primary school <9 years	0 (0)	0 (0)	1 (3.7)
	Primary school ≥9 years	1 (3.6)	0 (0)	1 (3.7)
	Secondary school ≤2 years	0 (0)	0 (0)	3 (11.1)
	Secondary school 3 years	2 (7.1)	2 (7.4)	3 (11.1)
	Other education <3 years	2 (7.1)	0 (0)	5 (18.5)
	Other education ≥3 years	1 (3.6)	1 (3.7)	1 (3.7)
	University <3 years	2 (7.1)	5 (18.5)	2 (7.4)
	University ≥3 years	20 (71.4)	19 (70.4)	11 (40.7)
Worry symptoms duration	Duration in years	19.6 (14.9)	18.2 (11.5)	19.4 (13.8)
Psychiatric diagnoses according to M.I.N.I.	Depression	8 (28.6)	4 (14.8)	8 (29.6)
	Panic Disorder	1 (3.6)	0 (0)	2 (7.4)
	Agoraphobia	0 (0)	0 (0)	2 (7.4)
	Social Anxiety Disorder	6 (21.4)	4 (14.8)	8 (29.6)
	Obsessive-compulsive disorder	1 (3.6)	3 (11.1)	1 (3.7)
	Alcohol Use Disorder	1 (3.6)	0 (0)	1 (3.7)
	Binge Eating Disorder	0 (0)	0 (0)	2 (7.4)
	Generalized Anxiety Disorder	18 (64.3)	13 (48.1)	15 (55.6)
	No diagnosis	6 (21.4)	8 (29.6)	7 (25.9)
Medication	Antidepressants	6 (21.4)	6 (22.2)	7 (25.9)
	Anxiolytics and/or sedatives	6 (21.4)	7 (25.9)	5 (18.5)
	Neuroleptics and/or anti-epileptics	1 (3.6)	1 (3.7)	0 (0)
	Central stimulants	2 (7.1)	0 (0)	3 (11.1)

### 2.3 Intervention

The intervention consisted of five modules (text chapters) which were accessed via an encrypted online platform. Each module contained a text section, a homework exercise, and at least one worksheet. Participants were encouraged to work with each module for approximately one week. Participants randomized to the intervention with therapist support could expect a response from a designated therapist within 36 h on weekdays. This response was provided in an email-like function in the online platform and contained mainly feedback on exercises, general attention, and support to progress with the program. Participants in the guided group were given consecutive access to the next module as they completed the previous one. The therapists were two master level students in psychology (the second and third author) who received on-demand supervision by the first and last author. Participants randomized to the unguided version of the intervention had full access to all modules from the start and were instructed to work at their own pace for five weeks. They were provided with a phone number to the study personnel in case of acute worsening of symptoms.

The intervention has previously been tested in a trial for excessive worry specifically related to the COVID-19 pandemic (Wahlund et al., 2021). In the current study, we expanded the content to be relevant for all types of worries and not specifically to COVID-19 themes. Given the broadened scope of the current intervention, we decided to extend the time frame from three to five weeks to accommodate participants' need to address multiple worrisome topics.

The first module provided participants with psychoeducation about worry and the difference between functional worries (worry that helps in everyday life) and dysfunctional worries (worry that does not help in everyday life). The participants were encouraged to keep a worry diary for a few days and label each worry thought as helpful or unhelpful. The second module provided the participants with a framework for how to cope with worrisome thoughts related to solvable problems. Here, the participants were encouraged to identify solvable worries in the diary and apply standard problem-solving techniques for these cognitions. The third module focused on excessive checking and why this behavior often leads to elevated levels of worry through negative reinforcement. The participants were subsequently encouraged to identify their own checking behaviors and try to limit or postpone these. In the fourth module, the participants were invited to practice techniques for detaching oneself from unsolvable, worrisome thoughts rather than mentally engaging with them. The fifth and final module provided the participants with a framework for how to engage in practices that would help shift focus from worries to other, more meaningful activities. The final module also contained a summary of the strategies provided in the program and a relapse prevention plan. More details about the intervention can be found in Wahlund et al. (2021). The mean number of completed modules were 3.6/5.

### 2.4 Waiting list

Participants randomized to the waiting list knew that they would receive the intervention after five weeks. They were provided with a telephone number to a study clinician if their symptoms worsened. No other information or support was provided to participants randomized to the waiting list.

### 2.5 Measurements and assessment points

#### 2.5.1 Primary outcome

The primary outcome was the GAD-7 (Spitzer et al., 2006), which assesses symptoms related to excessive worry and GAD during the past two weeks. It contains seven items rated on a four-point scale from 0 (not at all) to 3 (almost every day). GAD-7 has shown good validity and reliability, with measures of internal consistency from 0.88 to 0.92 and good test-retest reliability of *r* = 0.83 (Beard and Björgvinsson, 2014; Johnson et al., 2019; Spitzer et al., 2006). The GAD-7 was assessed each week during the intervention period of five weeks. The primary endpoint was set to week 5 (post intervention). Participants randomized to one of the intervention groups also completed a follow-up assessment seven weeks after the primary endpoint.

### 2.6 Secondary outcomes

The Work and Social Adjustment Scale (WSAS; Mundt et al., 2002) was used to measure daily functioning and was administered week 0–5. The PSWQ (Meyer et al., 1990) was used as a secondary measure of excessive worry. Intolerance of uncertainty has been suggested to be a core mechanism in the maintenance of worry (Morriss, 2025), and was therefore assessed using the Intolerance of Uncertainty Scale (IUS; Buhr and Dugas, 2002). Depression is common among individuals with excessive worry (Starcevic, 1995), and we therefore used depressive symptoms as treatment outcome with the MADRS-S (Svanborg and Åsberg, 2001). As insomnia is strongly linked to excessive worry, we wanted to assess if the intervention led to improved sleep. Here, we used the Insomnia Severity Index (ISI) as a measure of insomnia (Bastien et al., 2001). The IUS, MADRS-S and ISI were all administered at baseline and week 5. The frequency of adverse events was assessed week 1–5 with a self-report questionnaire, which has been used in a previous trial with similar results as face-to-face interviews (Andersson et al., 2015). Satisfaction with the intervention was assessed at week 5 using the Client Satisfaction Questionnaire (CSQ; Larsen et al., 1979). The PSWQ and the WSAS were also assessed seven weeks after completion of the primary endpoint assessment.

### 2.7 Statistical analyses

Between-group differences in symptom change over time were analyzed using a mixed effects regression framework (intention-to-treat) with random intercepts and random slopes in Stata 16.1. We analyzed intervention vs. waiting list and subsequently the two different formats (guided and unguided) vs. waiting list. Post-intervention effect sizes at week 5 were estimated using Cohen's *d*. Between-group differences (guided vs. unguided format) on the CSQ were tested using a linear regression analysis at the primary endpoint (week 5). Durability of improvements in the intervention groups was analyzed by investigating the effect of time on the GAD-7, PSWQ and the WSAS from the primary endpoint to the 7-week follow-up. As there were elevated data attrition at the 7-week follow-up, the analyses were repeated using multiple imputation.

We also investigated if reductions in symptoms of worry mediated improvements in daily functioning using the weekly scores on the WSAS. Direct, indirect, and total effects were estimated by obtaining the individual slope change on both the outcome and the mediator in the mixed effects regression framework using all weekly data on the GAD-7 and the WSAS. We used the medeff command in Stata 16.1 to estimate 95 % confidence intervals of the estimated effects (1000 replications) with robust standard errors (Hicks and Tingley, 2011; Imai et al., 2010a, Imai et al., 2010b). Given the relatively small sample size, we modelled a simultaneous mediation effect. We also conducted a sensitivity analysis to test to what extent the results may be affected by mediator-outcome confounding. This was done by fixing the residual correlation between the mediator and the outcome from *r* = −0.9 to 0.9 using the Medsens command in Stata (Hicks and Tingley, 2011; Imai et al., 2010a; Imai et al., 2010b).

## 3 Results

### 3.1 Intervention groups vs. waiting list

We first compared all participants randomized to the intervention (i.e. both guided and unguided intervention) vs. participants allocated to the waiting list. The mixed-effects regression analysis showed a significant larger reduction on the GAD-7 in the intervention group (*n* = 55) compared to the waiting list from week 0 to week 5 (*n* = 27; β = 0.76, Z = 4.29, *p* < .001) with a large between-group effect size at week 5 (*d* = 0.96). Significant interaction effects favoring the intervention were also seen on the WSAS (β = 0.93, Z = 3.13, *p* < .01), PSWQ (β = 6.61, Z = 4.03, p < .001), ISI (β = 2.32, Z = 2.71, *p* < .01) and the IUS-12 (β = 4.33, Z = 2.77, p < .01) but not on the MADRS-S (β = 2.20, Z = 1.83, *p* = .067). Mean scores with standard deviations are shown in Table 2.

**Table 2:** Outcome mean scores.

		Intervention (n = 55)		Waiting list (n = 27)		Guided intervention (n = 28)		Unguided intervention (n = 27)	
		M	SD	M	SD	M	SD	M	SD
GAD-7	Week 0	12.98	4.64	13.15	3.76	13.93	5.11	12.00	3.96
	Week 1	10.85	4.89	11.70	4.61	11.37	5.00	10.28	4.79
	Week 2	9.23	4.47	11.78	4.59	9.80	4.86	8.61	4.02
	Week 3	8.63	5.06	11.86	4.38	9.43	6.00	7.86	3.96
	Week 4	7.53	5.01	11.00	4.23	7.50	5.48	7.57	4.60
	Week 5	7.12	4.66	11.44	4.13	7.28	5.12	6.96	4.23
	FUP	6.56	4.07			7.15	5.48	6.00	2.00
WSAS	Week 0	15.93	8.16	16.33	8.90	17.57	8.35	14.22	7.52
	Week 1	14.00	7.37	14.70	7.08	14.41	7.64	13.56	7.19
	Week 2	12.17	8.20	14.70	8.41	13.04	8.94	11.22	7.40
	Week 3	11.51	8.72	15.23	9.63	12.86	10.02	10.22	7.28
	Week 4	10.14	8.88	15.63	9.24	10.05	9.88	10.24	7.94
	Week 5	11.71	9.00	16.89	8.89	12.50	9.59	10.82	8.22
	FUP	8.05	7.48			9.00	8.84	7.14	5.99
PSWQ	Week 0	67.15	7.67	67.52	7.42	69.54	7.82	64.67	6.79
	Week 5	59.62	9.15	66.00	7.41	59.29	10.82	59.77	6.92
	FUP	55.90	8.03			54.60	9.77	57.20	5.79
ISI	Week 0	13.11	7.16	12.70	6.24	14.57	6.84	11.59	7.30
	Week 5	10.40	6.61	12.41	5.96	11.42	6.59	9.36	6.46
MADRS-S	Week 0	18.84	7.89	21.30	6.06	20.46	7.79	17.15	7.78
	Week 5	15.11	8.18	19.67	5.63	15.88	8.85	13.91	7.44
IUS-12	Week 0	40.20	9.27	39.74	8.92	41.96	9.11	38.37	9.26
	Week 5	36.31	8.82	40.00	10.69	35.71	10.14	36.50	7.42

Abbreviations: GAD-7, Generalized Anxiety Disorder 7-items scale; WSAS, Work and Social Adjustment Scale; PSWQ, Penn State Worry Questionnaire; ISI, Insomnia Severity Index; MADRS-S, Montgomery Asberg Depression Rating Scale – Self report; IUS-12, Intolerance of Uncertainty Scale 12 items; FUP, Follow-up.

### 3.2 Guided- and unguided Intervention vs. waiting list

Secondly, we compared the guided and unguided groups separately to the waiting list group. Participants randomized to the guided intervention with therapist support had significant improvements on the GAD-7 (β = 0.92, Z = 4.29, p < .001), WSAS (β = 1.07, Z = 3.27, p < .01), PSWQ (β = 8.76, Z = 4.66, p < .001), ISI (β = 3.18, Z = 3.10, p < .01), MADRS-S (β = 3.11, Z = 2.31, *p* < .05) and the IUS-12 (β = 6.85, Z = 3.85, p < .001). The between group effect size on the GAD-7 at week 5 was large (*d* = 0.90). At the 7-week follow-up, participants in the guided intervention group had sustained results on the GAD-7 (β = 0.32, Z = 0.38, *p* = .70) and further improvements on the PSWQ (β = 3.16, Z = 1.96, *p* = .05) and WSAS (β = 2.96, Z = 3.15, p < .01). Estimates from the multiple imputation of week 7 scores were largely the same as in the main analysis (GAD-7: β = 0.46, *t* = 0.39, p = .70; PSWQ: β = 4.72, *t* = 2.07, p < .05; WSAS: β = 3.36, *t* = 1.86, p = .06).

Results were largely similar for individuals randomized to the unguided version of the intervention with significant reductions on the GAD-7 (β = 0.59, Z = 3.26, p < .01), WSAS (β = 0.77, Z = 2.33, p < .05), and PSWQ (β = 4.32, Z = 2.88, p < .01), but not on the ISI (β = 1.36, Z = 1.62, *p* = .11), the MADRS-S (β =1.18, Z = 1.05, *p* = .29) and the IUS-12 (β = 1.60, Z = 1.09, *p* = .27). The between-group effect size on the GAD-7 at week 5 was also in the large range (*d* = 1.07). The 7-week follow-up assessment of the unguided intervention group showed sustained results on the GAD-7 (β = −0.57, Z = 0.98, *p* = .33) and on the PSWQ (β = −2.20, Z = 1.46, *p* = .15) with further improvements on the WSAS (β = −3.07, Z = 2.18, p < .05). Multiple imputation showed similar results (GAD-7: β = 1.02, *t* = 1.00, *p* = .32; PSWQ: β = 3.29, *t* = 1.67, *p* = .095; WSAS: β = 3.14, *t* = 2.10, p < .05).

### 3.3 The impact of reduced worry on improvements in daily functioning

**Fig. 2:**
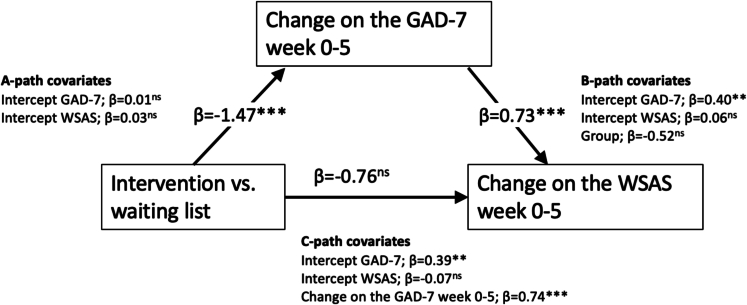
Mediation effects Abbreviations: GAD-7, Generalized Anxiety Disorder 7-items scale; WSAS, Work and Social Adjustment Scale.

**Fig. 3:**
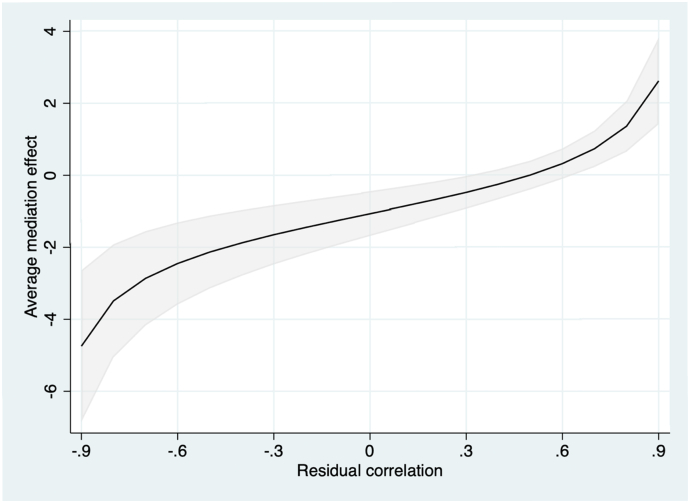
Sensitivity analysis of mediation effects.

The mediation model indicated a significant effect of group allocation and reductions in worry and a significant relationship between reductions in worry and improvements in daily functioning (Fig. 2). The total effect (i.e. the overall effect of group on the outcome [daily functioning]) was significant (estimate −1.58; 95 % CI -0.54 – -2.53). The direct effect (i.e. the effect of group on the outcome [daily functioning] accounting for the variance of the mediator [reductions in excessive worry]) was not significant (estimate −0.51; 95 % CI 0.48 – −1.37 while the indirect effect (i.e. the effect of group on the outcome [daily functioning] through the mediator [reductions in excessive worry]) was significant (estimate −1.06; 95 % CI -0.51 – −1.76) with 66 % of the effects being attributed to the mediator. The sensitivity analysis indicated that the mediation effects were fairly robust to unmeasured mediator-outcome confounders allowing for a significant mediation effect given a residual correlation of *r* = 0.3 (Fig. 3).

### 3.4 Intervention satisfaction and adverse events

A regression analysis did not show a significant difference on the CSQ at week 5 (β = 2.05, *t* = 1.95, *p* = .058) but participants randomized to the guided intervention format scored generally somewhat higher than participants randomized to unguided format on each individual item (see Table 3 for details). The guided intervention recorded the following weekly adverse events; increased worry (9 reports), sleep problems (2), increased pain sensitivity (3), tenseness (1), tiredness (1), and concentration difficulties (1). For the unguided intervention, the following adverse events were recorded; increased worry (10 reports), sleep difficulties (1), depressive symptoms (3), and increased stress (7). All adverse events were rated as being transient and in the mild range.

**Table 3:** Response frequency on each item on the CSQ.

How would you rate the quality of service you received?	Guided intervention	Unguided intervention
Excellent	12 (52 %)	7 (32 %)
Good	10 (43 %)	13 (59 %)
Fair	1 (4 %)	2 (9 %)
Poor	0 (0 %)	0 (0 %)

Did you get the kind of service you wanted?		
No, definitely not	0 (0 %)	0 (0 %)
No, not really	5 (22 %)	6 (27 %)
Yes, generally	11 (48 %)	14 (64 %)
Yes, definitely	7 (30 %)	2 (9 %)

To what extent has our service met your needs?		
Almost all of my needs have been met	7 (30 %)	1 (5 %)
Most of my needs have been met	7 (30 %)	8 (36 %)
Only a few of my needs have been met	9 (39 %)	12 (55 %)
None of my needs have been met	0 (0 %)	1 (5 %)

If a friend were in need of similar help, would you recommend our service to him or her?		
No, definitely not	0 (0 %)	0 (0 %)
No, I don't think so	2 (9 %)	2 (9 %)
Yes, I think so	8 (35 %)	13 (59 %)
Yes, definitely	13 (57 %)	7 (32 %)

Have the services you received helped you to deal more effectively with your problems?		
Yes, they helped a great deal	11 (48 %)	3 (14 %)
Yes, they helped somewhat	11 (48 %)	17 (77 %)
No, they really didn't help	1 (4 %)	2 (9 %)
No, they seemed to make things worse	0 (0 %)	0 (0 %)

In an overall, general sense, how satisfied are you with the service you received?		
Very satisfied	12 (52 %)	6 (27 %)
Mostly satisfied	8 (35 %)	15 (68 %)
Indifferent of mildly dissatisfied	3 (13 %)	1 (5 %)
Quite dissatisfied	0 (0 %)	0 (0 %)

If you were to seek help again, would you come back to our service?		
No, definitely not	0 (0 %)	0 (0 %)
No, I don't think so	0 (0 %)	2 (9 %)
Yes, I think so	8 (35 %)	10 (45 %)
Yes, definitely	15 (65 %)	10 (45 %)

Abbreviation: CSQ, Client Satisfaction Questionnaire.

## 4 Discussion

This study showed that a brief online intervention produced large reductions in excessive worry against a waiting list control group. Both guided and unguided formats showed large between-group effect sizes at week 5 compared to the waiting list. Reductions in worry, in turn, mediated improvements in daily functioning. The sensitivity analysis indicated that the mediation effects were fairly robust to mediator-outcome confounding, with significant mediation effects given a residual correlation between the mediator and the outcome of *r* = 0.3. There were few recorded adverse events and intervention satisfaction was generally high.

Previous research has shown that individuals with GAD account for about half of treatment-seeking patients with anxiety disorders in primary care. A substantial additional portion of the primary care population presents with subthreshold symptoms of GAD and has significant impairments equal to full-blown GAD (Haller et al., 2014; Wittchen and Hoyer, 2001). The results from the mediation analysis in the present study are clinically important as they indicate that it is possible to improve daily functioning in high-worriers by delivering a brief online intervention that specifically targets excessive worry, irrespective of primary diagnosis. This might be particularly relevant for primary care clinicians who often struggle to provide patients with full-dose disorder-specific treatments (Wakida et al., 2018). One suggestion for future research could be to provide treatment-seeking patients with excessive worry in primary care contexts the unguided version of the intervention and gradually step up the dosage of therapist input for individuals who are at risk of not achieving beneficial effects during the intervention (e.g. Forsell et al., 2019) or to provide full dose face-to-face treatments for those who do not respond to the first treatment attempt (e.g. Salomonsson et al., 2018). This suggested stepped-care approach could further improve the effectiveness while ensuring that therapist resources are allocated to patients in most need of additional therapist support. This approach could also be expanded to other worry-related problems that can be effectively treated with psychological interventions.

Almost one fourth of the sample in the present study did not fulfill diagnostic criteria for any common psychiatric disorder but struggled with excessive worry only. Previous research has indicated that excessive worry is a risk factor for developing other, more serious mental health problems (S. J. Davies et al., 2016; Hinton et al., 2011). Therefore, it is important to investigate whether this intervention could help prevent the development of mental health problems or prevent already existing psychiatric symptomatology from deteriorating or becoming chronic. For instance, previous research has indicated that excessive worry has a negative effect on sleep quality (Jansson and Linton, 2006; Tang and Harvey, 2004). An important next step could be to test longitudinally if reductions in worry lead to subsequent improvements in sleep quality and/or prevent the development of insomnia later in life. Another mental health disorder that has been shown to be highly affected by worry is PTSD (Butler et al., 1995; Hinton et al., 2011). Here, it would be interesting to investigate if reduced worry from an unguided low-threshold intervention could reduce the risk of developing PTSD following a traumatic event.

This study comes with limitations. First, we did not conduct long-term follow-up assessments beyond seven weeks, which makes the longevity of the intervention effects hard to determine. A previous mediation analysis by our research group showed that reductions in worry due to the intervention were associated with improved daily functioning even one year after intervention completion (Andersson et al., 2024). To establish the benefits of low-threshold worry interventions over time, more long-term follow-ups are needed. Second, we used self-administered outcome measures, which in a meta-analysis have been shown to estimate lower effect sizes than clinician-rated instruments (Cuijpers et al., 2014). Consequently, it is possible that the estimated effect sizes in this study were underestimated. A third important limitation in this study is that we used a parallel mediation analysis due to the relatively low degree of power. This analysis limits the ability to determine the time-specific causality between reductions in excessive worry and improvements in daily functioning (Maxwell and Cole, 2007). One recommended next step would therefore be to conduct a trial with the aim of directly manipulating the hypothesized mediator of change (e.g. participants randomized to three different interventions with low, average, and high degrees of efficacy in reducing worry) and to test if this in turn leads to differential improvements in daily functioning. Furthermore, the power to detect effects in the mediation analyses was modest (52 %), which increases the risk of Type II errors and limits the robustness of the conclusions. Therefore, the results of the mediation analyses should be interpreted with caution. Future studies with larger sample sizes are needed to more reliably assess potential mediators of treatment outcome. A final limitation lies in the rather well-educated sample which could have affected the generalizability of the findings. Future trials should investigate if this type of intervention is also effective in a hospital setting for individuals with more severe symptoms.

Altogether, this study shows that it is feasible to provide a brief online intervention for high-worriers and that reductions in worry seem to drive improvements in daily functioning. More research is needed to identify for whom this kind of intervention works and if reductions in worry lead to other positive knock-on effects, such as a lower risk of developing subsequent mental health problems.

## CRediT authorship contribution statement

EA was the principal investigator of the trial. TW and EA designed the trial. TW and EA took the main responsibility for drafting the manuscript of the current study. EA did the statistical analyses. TW, EA, VV and FS collected all trial data. All authors contributed to, read, and approved the final report. The lead author attests that all listed authors meet authorship criteria and that no others meeting the criteria have been omitted.

## Statement of ethics

The authors assert that all procedures contributing to this work comply with the ethical standards of the relevant national and institutional committees on human experimentation and with the Helsinki Declaration of 1975, as revised in 2008*.*

## Declaration of competing interest

EA and TW have co-authored a self-help book for excessive worry which is based on the current online intervention.

## Data Availability

The raw data supporting the conclusions of this article will be made available by the authors upon request given that the request complies with Swedish and EU laws regulating the protection of identifiable data.
